# Plant defensive responses to insect eggs are inducible by general egg-associated elicitors

**DOI:** 10.1038/s41598-024-51565-y

**Published:** 2024-01-11

**Authors:** Vivien Lortzing, Georgios Valsamakis, Friederike Jantzen, Janik Hundacker, Luis R. Paniagua Voirol, Fabian Schumacher, Burkhard Kleuser, Monika Hilker

**Affiliations:** 1 Applied Zoology/Animal Ecology, Institute of Biology, Dahlem Centre of Plant Sciences, Freie Universität Berlin, Haderslebener Str. 9, 12163 Berlin, Germany; 2 Microbiology, Institute of Biology, Dahlem Centre of Plant Sciences, Freie Universität Berlin, Königin-Luise-Str. 12-16, 14195 Berlin, Germany; 3 Pharmacology and Toxicology, Institute of Pharmacy, Freie Universität Berlin, Königin-Luise-Str. 2-4, 14195 Berlin, Germany; 4 Core-Facility BioSupraMol, PharmaMS Subunit, Institute of Pharmacy, Freie Universität Berlin, Königin-Luise-Str. 2-4, 14195 Berlin, Germany

**Keywords:** Chemical biology, Ecology, Physiology, Plant sciences, Zoology

## Abstract

Egg deposition by herbivorous insects is well known to elicit defensive plant responses. Our study aimed to elucidate the insect and plant species specificity of these responses. To study the insect species specificity, we treated *Arabidopsis thaliana* with egg extracts and egg-associated secretions of a sawfly (*Diprion pini*), a beetle (*Xanthogaleruca luteola*) and a butterfly (*Pieris brassicae*). All egg extracts elicited salicylic acid (SA) accumulation in the plant, and all secretions induced expression of plant genes known to be responsive to the butterfly eggs, among them *Pathogenesis-Related (PR)* genes. All secretions contained phosphatidylcholine derivatives, known elicitors of SA accumulation and *PR* gene expression in *Arabidopsis.* The sawfly egg extract did not induce plant camalexin levels, while the other extracts did. Our studies on the plant species specificity revealed that *Solanum dulcamara* and *Ulmus minor* responded with SA accumulation and cell death to *P. brassicae* eggs, i.e. responses also known for *A. thaliana.* However, the butterfly eggs induced neoplasms only in *S. dulcamara.* Our results provide evidence for general, phosphatidylcholine-based, egg-associated elicitors of plant responses and for conserved plant core responses to eggs, but also point to plant and insect species-specific traits in plant–insect egg interactions.

## Introduction

Plants are exposed to a multitude of biotic stressors, including infection by phytopathogens and attack by herbivorous insects. As sessile organisms, plants have evolved finely adjusted defence strategies to protect themselves from biotic stress^1–5^. Their defences do not only target the feeding herbivore itself, but also the eggs laid by an herbivorous insect onto the leaves.

Plant defence against insect eggs is a preventive strategy and acts even before the plant is damaged by hatching larvae. Constitutive defences against eggs are e.g. leaf trichomes, deterrents or lack of oviposition stimulants that prevent herbivorous insects from egg deposition^6–8^. In addition, plants can mobilize defences in response to egg deposition. Such inducible plant responses to insect eggs can directly harm the eggs or act indirectly for defence by attracting and arresting parasitoids that kill the eggs^9–14^.

Indirect plant defences induced by insect eggs especially rely on oviposition-induced plant volatiles that attract egg parasitoids^9,15–17^. Egg deposition can also induce changes in leaf surface chemistry, which result in more efficient and intense foraging behaviour of egg parasitoids^18,19^.

Egg-induced, direct plant defences can be effective by producing ovicides that kill the eggs or by forming new plant tissue that crushes the eggs; furthermore, plants can respond to insect eggs by detaching them from the plant or by desiccating them^9,10,12^. Plants can detach eggs from leaves by formation of neoplasms that lift the eggs from the leaf surface so that they easily fall off^20,21^. In addition, the formation of necrotic tissue at the site of egg deposition by hypersensitive-response (HR)-like changes can lead to detachment of eggs or egg desiccation^22–24^. The formation of egg-induced HR-like necrosis and chlorosis is described for different plant families, including Brassicaceae, Solanaceae, Fabaceae and Pinaceae, and in response to different insect species, including different insect orders like Lepidoptera, Coleoptera and Hymenoptera^11,25,26^.

Several elicitors of plant responses to insect eggs have been identified from egg extracts, from extracts of female insects and from exocrine egg-associated secretions, which attach the eggs to the leaf surface and thus, are in immediate contact with the leaf ^27^. The chemical identity of these elicitors varies with the insect species studied (Table 1).

**Table 1:** Chemically identified insect egg-associated elicitors of plant defence responses.

Insect species (Order)	Plant species	Elicitor	Elicitor identified from	Plant response to elicitor	References
*Pieris brassicae* (Lepidoptera)	*Arabidopsis thaliana*	Phosphatidylcholine derivatives (PCs)	Egg extract	Accumulation of SA Induction of *PR1*	^45^
	*Arabidopsis thaliana* *Brassica oleracea* var. *gemmifera*	Benzyl cyanide	Egg secretion provided by accessory reproductive gland of mated females	Change in leaf surface chemistry; arrestment of egg parasitoids	^18^ ^65^
*P. rapae* (Lepidoptera)		Indole	Egg secretion provided by accessory reproductive gland of mated females	Arrestment of egg parasitoids	^81^
*Sogatella furcifera* (Hemiptera)	*Oryza sativa*	Phosphatidylcholine derivatives (PCs)	Female extract	Production of ovicidal plant compound	^82,83^
*Bruchus pisorum* *Callosobruchus maculatus* (Coleoptera)	*Pisum sativum* (*Np* pea line)	Bruchins (long-chain α,ω-diols mono- or di-esterified with 3-hydroxypropanoic acid)	Female extract	Formation of neoplasms	^20^
*Diprion pini* (Hymenoptera)	*Pinus sylvestris*	Diprionin (annexin-like protein)	Egg secretion provided by the glandular cells of the oviduct	Change in pine odour (attraction of egg parasitoids) Change in expression of defence genes	^64^

Several plant species have been studied with respect to the ecological effects of their responses to insect egg depositions^9^. However, the mechanisms of egg-induced responses have been investigated till now only in a few plant species^13,28,29^. While even closely related brassicaceous host plant species of the butterfly *Pieris brassicae* (L.) were found to differ in their responses to eggs of this butterfly species^26,29^, a comparison of egg-induced responses of Brassicaceae, Solanaceae and Ulmaceae species revealed some commonalities with respect to the chemical, phytohormonal and transcriptional responses to eggs. Some of the detected similarities are independent of the oviposition mode of the insect species. For example, regardless of whether eggs are laid in clusters or singly, or whether oviposition is associated with leaf wounding or not, several plant species were shown to enhance their concentrations of leaf phenylpropanoids when egg deposition is followed by larval feeding^28,30^. Furthermore, several plant species are known to accumulate reactive oxygen species (ROS) at the site of insect egg deposition^26,29,31–34^. Accumulation of hydrogen peroxide can directly kill insect eggs ^34^. Accumulation of ROS can also elicit HR-like symptoms^26,29,32–34^, which are known to be also formed after infection of plants with (hemi)biotrophic phytopathogens^35,36^.

Indeed, plant responses to insect eggs are well known to show several parallels to plant responses to (hemi)biotrophic phytopathogens^37,38^. For example, thale cress *Arabidopsis thaliana* (L.) Heynh. accumulates the phytoalexin camalexin in response to phytopathogens^39^, but also in response to *P. brassicae* eggs^40,41^. Furthermore, *A. thaliana* leaves with *P. brassicae* eggs accumulate H_2_0_2_ and salicylic acid (SA) and increase their transcript levels of genes encoding typical pathogenesis-related (PR) proteins^32,41–44^. Enhanced expression of *PR1* as well as accumulation of SA were also shown in response to treatment of *A. thaliana* with phosphatidylcholine derivatives (PCs) that have been isolated from *P. brassicae* egg extracts (Table 1)^45^. A secretion from the accessory reproductive glands of *P. brassicae* females is also known to induce *PR1* and *PR5* in *A. thaliana*^43^*.*

Many of the currently available studies have been conducted with one plant species responding to eggs of one particular insect species that uses the studied plant species as food plant. Therefore, in spite of our knowledge about commonalities in the responses of some plant species to insect eggs, only little knowledge is available on the insect species specificity of plant responses to eggs of different insect species. For example, a recent study by Caarls et al*.*^29^ showed that eggs of the specialist *P. brassicae* and the generalist *Mamestra brassicae* (L.) elicit the accumulation of ROS in the host plant *Brassica nigra,* but the plant responses to eggs of these two lepidopteran species differed with respect to *PR1* expression, cell death and ethylene emission. Similarly, HR-like symptoms were found to be induced in *Brassica nigra* (L.) by eggs of some members of the pierid butterfly taxon, but not by all^26^. If eggs laid by different insect species onto a plant elicit similar responses, a common, 'general' insect egg elicitor of these responses might be responsible.

Our study aimed to further deepen the knowledge about the species specificity of plant responses to insect eggs. We chose species, for which egg-induced plant defensive responses are known and which belong to different taxonomic categories. The studied plant species range from *A. thaliana* (Brassicaceae) to bittersweet nightshade (*Solanum dulcamara* L., Solanaceae) and elm (*Ulmus minor* Mill.*,* Ulmaceae). The insect species studied were *P. brassicae* (Pieridae, Lepidoptera), the sawfly *Diprion pini* (L.) (Diprionidae, Hymenoptera) and the elm leaf beetle *Xanthogaleruca luteola* Müller (Chrysomelidae, Coleoptera). Several egg-induced responses of *A. thaliana* to *P. brassicae* eggs have been mentioned above. Bittersweet nightshade responds to moth eggs by accumulation of H_2_0_2_, formation of chlorotic tissue, cell death and neoplasms at the site of oviposition^34^. Elm leaves change their odour in response to eggs and egg-associated secretion of the elm leaf beetle, thereby attracting egg parasitoids^46^. This plant and insect species spectrum allowed us to investigate interactions beyond those between insect eggs and the host plants of these insects. By this approach, we aimed to shed some light on possibly phylogenetically conserved plant responses to insect eggs as well as to possibly conserved, general plant response-eliciting compounds associated with insect eggs.

In detail, we addressed the following questions.

1. How insect species-specific is *A. thaliana* responding to egg extracts and egg secretions of different insect species?
2. How insect species-specific is the presence of PCs in insect egg secretions? We analyzed whether egg secretions of all the three insect species studied contain PCs, which are known to induce responses in *A. thaliana* that resemble the responses to *P. brassicae* eggs (i.e. accumulation of SA, induction of *PR1*^45^).
3. How plant species-specific are the responses to *P. brassicae* eggs? We measured the SA concentrations of non-host plants of *P. brassicae,* i.e. of *S. dulcamara* and *U. minor,* and tested these plants for H_2_O_2_ accumulation and cell death at the site of egg deposition by this butterfly.

## Results

### Induction of SA accumulation in *A. thaliana* by egg extracts is independent of the insect species, but induction of camalexin accumulation differs by the tested insect species

**Figure 1:**
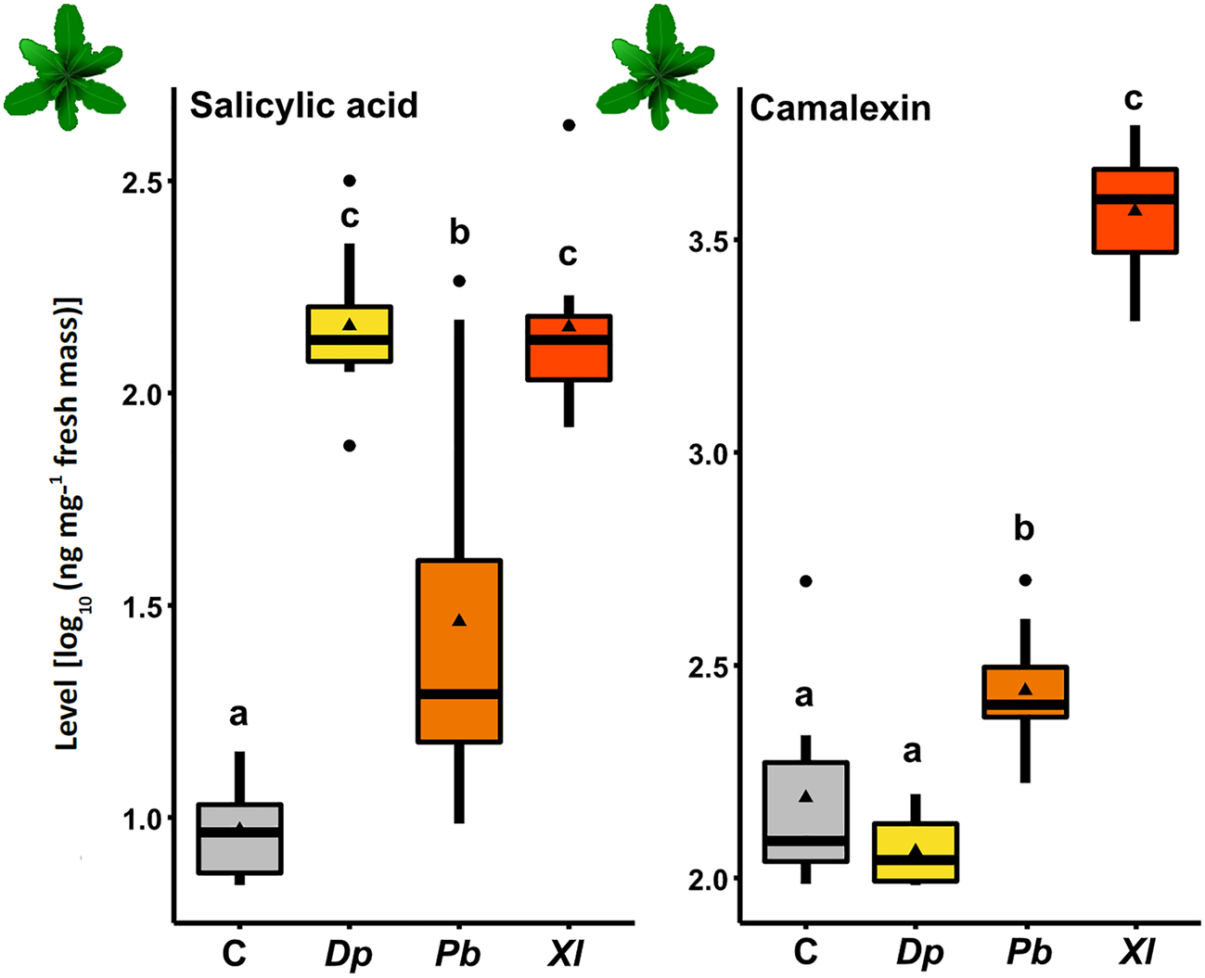
Response of *Arabidopsis thaliana* to egg extracts from *Diprion pini (Dp)*, *Pieris brassicae (Pb)* and *Xanthogaluerca luteola (Xl)* compared to response levels in leaves of untreated control (C) plants. Levels of salicylic acid and camalexin were determined. Boxplots show interquartile ranges (25–75%), medians (horizontal line), whiskers (10th and 90th percentiles), outliers (dots outside the 10th and 90th percentiles) and means (triangles) of log_10_-transformed salicylic acid and camalexin levels (ng per mg leaf fresh mass). Different letters indicate significant differences between treatments (*P* < 0.05, ANOVA with Tukey test post hoc). *N* = 8. Statistical details are provided in Supplementary Table S1.

To test the insect species specificity of *A. thaliana*'s responses to insect egg extracts, we quantified SA and camalexin in *A. thaliana* after treating the leaves with egg extract from a lepidopteran species (the butterfly* P. brassicae, Pb*), a hymenopteran species (the sawfly *D. pini, Dp)* and a coleopteran species (the beetle *X. luteola, Xl)* (Fig. 1).

The SA levels were significantly induced in response to egg extracts of all three insect species. Our analyses confirmed previous results, which showed that *Pb* egg extract induces SA in *A. thaliana*^42^. Similarly, the *Dp* and *Xl* egg extracts induced SA in *A. thaliana*, and, conspicuously, even stronger than *Pb* egg extract did (Fig. 1, Supplementary Table S1).

Furthermore, *A. thaliana* accumulated camalexin in locally treated leaves in response to application of *Pb* egg extract, as has already previously been shown by Alfonso et al*.*^41^. *Dp* egg extract did not induce camalexin, whereas *Xl* egg extract strongly induced camalexin in *A. thaliana*, even stronger than the *Pb* egg extract did (Fig. 1, Supplementary Table S1).

The responses of *A. thaliana* to *Pb* egg extracts shown here and in previous studies match the responses of this plant species to natural *Pb* egg deposition, which also induces SA and camalexin accumulation^40–42,47^.

Taken together, egg extracts from three insect species induced SA accumulation in *A. thaliana*, but exerted insect species-specific effects on the induction of camalexin.

### Egg secretions from a butterfly, a sawfly and a beetle induce defence genes in *A. thaliana*

**Figure 2:**
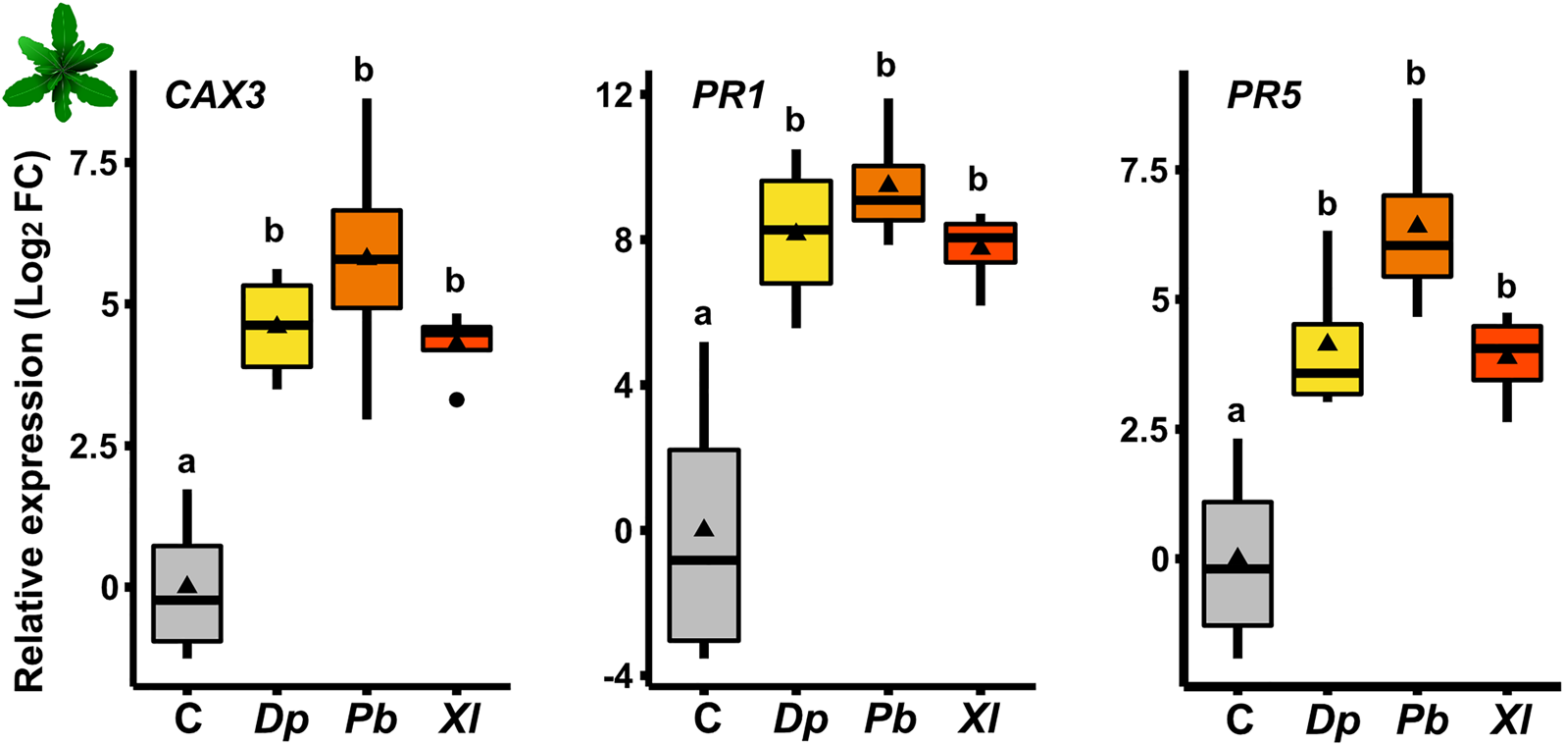
Response of *Arabidopsis thaliana* to egg secretions obtained from one individual of *Diprion pini (Dp)*, *Pieris brassicae (Pb)* and *Xanthogaluerca luteola (Xl)* compared to response levels in leaves of untreated control (C) plants*.* We determined expression levels of *CATION EXCHANGER 3 (CAX3)* and *PATHOGENESIS-RELATED- (PR)* genes *PR1* and *PR2.* Boxplots show interquartile ranges (25–75%), medians (horizontal line), whiskers (10th and 90th percentiles) and means (triangles). Expressions were normalized to two reference genes and untreated control leaves. Depicted are mean log_2_FC relative expressions. Different letters indicate significant differences between the treatments (*P* < 0.05, ANOVA with Tukey test post hoc). *N* = 4. Statistical details are provided in Supplementary Table S2.

In *A. thaliana* and other brassicaceous plants*,* the genes *CAX3*, *PR1* and *PR5* are typically responsive to *Pb* eggs, *Pb* egg secretions and egg extracts^26,42,43,47–49^. To test the insect species specificity of *A. thaliana*'s responses to insect egg secretions, we analyzed whether these genes are also inducible by egg secretions of *Dp* and *Xl.* We quantified the expression levels of these genes three days after application of the *Dp* and *Xl* egg-associated secretions to *A. thaliana* leaves. For comparison, we also applied *Pb* egg secretion (Fig. 2).

The expression of these three *A. thaliana* genes was significantly induced in response to all secretions independent from the insect species. Application of the egg secretions of *Dp* and *Xl* resulted in slightly weaker induction of the marker genes, however, the transcript levels were not significantly different from those induced by *Pb* egg secretion (Fig. 2, Supplementary Table S2).

Hence, *A. thaliana* showed insect species-unspecific responses to egg secretions with respect to the expression of several defence genes.

### Phosphatidylcholines are present in the egg-associated secretions of a butterfly, a sawfly and a beetle

We tested whether phosphatidylcholines (PCs) are present in egg-associated secretions of *Pb, Dp* and *Xl.* We focused on those PCs that have previously been isolated from *Pb* egg extracts and were shown to elicit plant responses similar to those of natural *Pb* egg deposition^45,50^, i.e. PCs with C16:1, C18:1, C18:3 fatty acyl chains.

**Figure 3:**
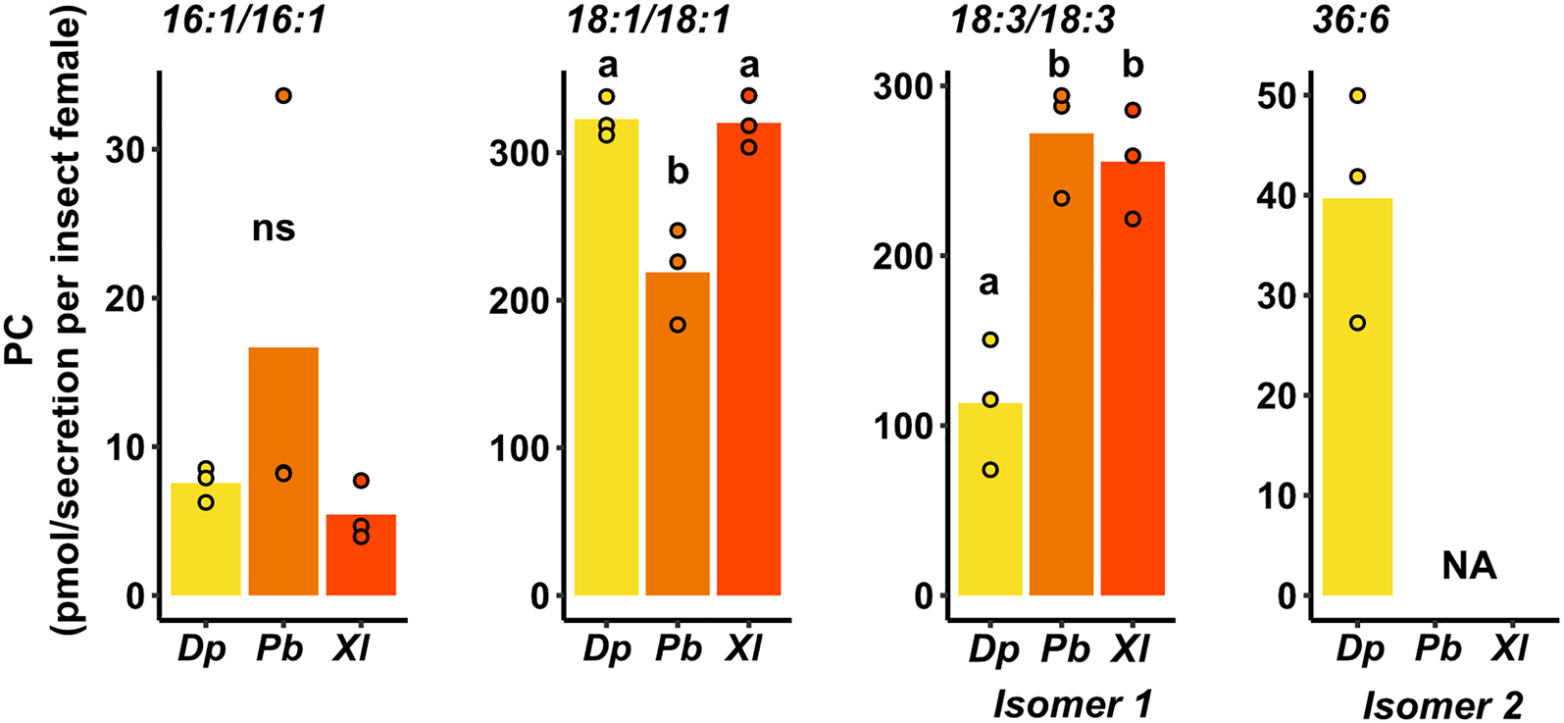
Phosphatidylcholines (PC 16:1/16:1, PC 18:1/18:1, PC 18:3/18:3 isomer 1 and isomer 2 (PC 36:6)) in egg secretions of *Diprion pini* (*Dp), Pieris brassicae* (*Pb*) and *Xanthogaleruca luteola* (*Xl).* Bars represent means and dots the data points. Different letters indicate significant differences of PC isomer quantities between species (*P* < 0.05, ANOVA with Tukey test post hoc), ns indicates non-significant differences between species for PC 16:1/16:1 (*P* > 0.05, Kruskal–Wallis test), and NA indicates that the isomer was not detected in *Pb* and *Xl* secretions (no statistical test applied). *N* = 3, per sample analyzed, we pooled the secretion from three individual female insects. Quantities given are calculated per egg secretion obtained from one *Dp* or *Xl* oviduct or one female *Pb* accessory reproductive gland. Statistical details are provided in Supplementary Table S3.

Our analyses revealed that *Pb* egg secretion, which is produced in the accessory reproductive glands of the female butterflies, contains these elicitor-active PCs (Fig. 3, Supplementary Fig. S1). Hence, these compounds that had previously been identified from egg extracts^45^, are also present in *Pb* egg secretion, which is in immediate contact with the leaves.

In addition, we tested whether these PCs are present in the secretions released from oviducts of *Dp* and *Xl,* i.e., secretions that encase the deposited eggs. The PC isomers (C16:1/C16:1, C18:1/C18:1, and C18:3/C18:3) were also present in the *Dp* and *Xl* egg-associated secretions (Fig. 3, Supplementary Fig. S1). The quantity of PC 18:1/18:1 was significantly lower in *Pb* secretion than in *Dp* and *Xl* secretions, while less PC 18:3/18:3 was detected in *Dp* secretion than in *Pb* and *Xl* secretions (Fig. 3, Supplementary Table S3). For the *Dp* secretion, we found an additional 36:6 PC isomer 2, which was absent in secretions from *Pb* and *Xl* (Fig. 3, Supplementary Figs. S1, Figs. S2). High-resolution and tandem-mass spectrometry proved that this detected isomer 2 must be an isomeric structure of the PC 18:3/18:3 isomer 1 present in the secretion of all three studied species. The MS/MS fragmentation pattern clearly indicated a phosphocholine group present in the molecule, which basically narrows down the possible lipid classes to PCs and sphingomyelins. The 18:3/18:3 isomer 1 present in the secretions of all three studied species, on the other hand, was clearly identified as PC 18:3(9*Z*,12*Z*,15*Z*)/18:3(9*Z*,12*Z*,15*Z*) via retention time using a reference standard. A Lipid Maps® search for the identity of the 36:6 PC isomer 2 exclusively detected in *D. pini* egg secretion revealed 16 possible isomeric PC subspecies that could match the exact mass-to-charge ratio of this isomer (Supplementary Fig. S2). Further analysis with additional reference standards is needed to clarify the structure of this isomer.

Taken together, the presence of PC*s* in egg-associated secretions is unspecific for the three studied insect species.

### *Solanum dulcamara*'s and *Ulmus minor*'s responses to butterfly eggs show similarities to *A. thaliana*'s responses to these eggs

To address the question how plant species-specific the responses of *A. thaliana* to *Pb* eggs are, we motivated *Pb* butterflies to lay egg clutches on the underside of the leaves of the non-host plants *S. dulcamara* (two genotypes) and *U. minor.* We could motivate the butterflies to oviposit on these leaves by placing a host plant leaf on top of the non-host plant leaves. We tested the response of these plants with respect to macroscopically visible changes, H_2_O_2_ accumulation (visualized by DAB staining), cell death (visualized by trypan blue staining) and SA accumulation at the site of egg deposition.

**Figure 4:**
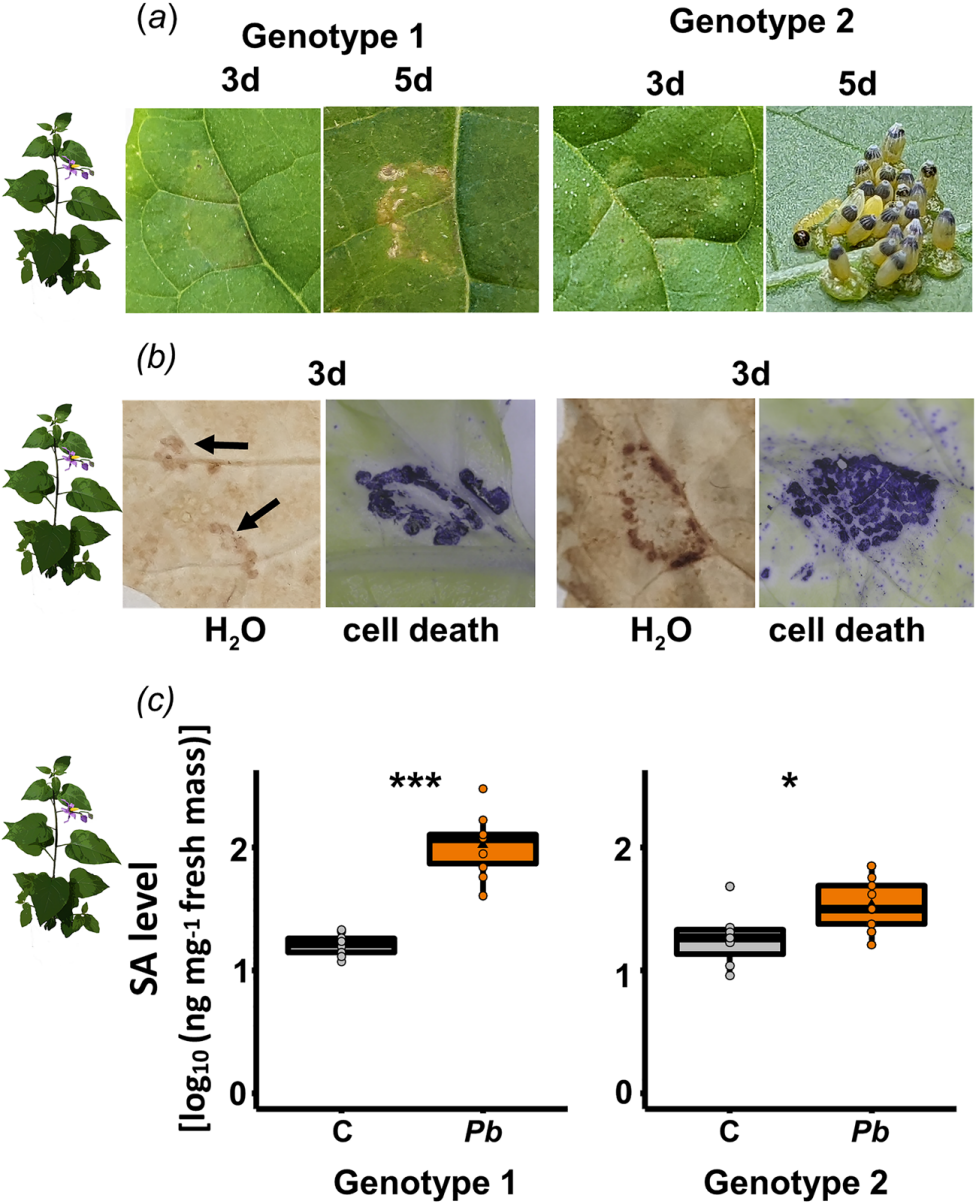
Response of two *Solanum dulcamara* genotypes to *Pieris brassicae* egg deposition. (**a**) Formation of chlorotic tissue underneath the eggs three days (3d) after deposition in both genotypes; formation of necrosis (genotype 1) and neoplasms (genotype 2) five days after deposition of the eggs (5d). (**b**) H_2_O_2_ and cell death (detection with 3,3-diaminobenzidine and trypan blue, respectively) in leaves underneath the eggs. (**c**) Levels of salicylic acid (SA) in leaves of *S. dulcamara* genotypes three days after *P. brassicae* egg deposition (*Pb*) compared to untreated control (C) leaves. Boxplots show interquartile ranges (25–75%), medians (horizontal line), whiskers (10th and 90th percentiles) and means (triangles) of log_10_-transformed SA level (ng per mg leaf fresh mass). Dots represent all data points. Asterisks (*/***) indicate significant differences between treatments (*P* < 0.05/ 0.001, Student’s *t*-test). Genotype 1 *N* = 10, genotype 2 *N*_C_ = 7, *N*_E_ = 9. Statistical details are provided in Supplementary Table S4.

Both *S. dulcamara* genotypes showed macroscopically visible, light greenish, i.e. chlorotic tissue underneath the egg clutch three days after *Pb* egg deposition (Fig. 4a). Application of *Pb* egg extracts also resulted in chlorotic tissue formation in genotype 2 (Supplementary Fig. S3). Five days after egg deposition, briefly before the larvae hatch, genotype 1 had formed brownish, necrotic tissue underneath the egg clutch, and genotype 2 had also started to form neoplasms at the site where the eggs had been laid (Fig. 4a).

Staining of the oviposition sites with DAB revealed that both *S. dulcamara* genotypes respond to *Pb* eggs with accumulation of H_2_O_2_ (Fig. 4b). Furthermore, for both genotypes we could visualize leaf cell death with trypan blue staining at the oviposition site (Fig. 4b). Egg deposition by *Pb* induced SA concentrations in both *S. dulcamara* genotypes (Fig. 4c, Supplementary Table S4).

**Figure 5:**
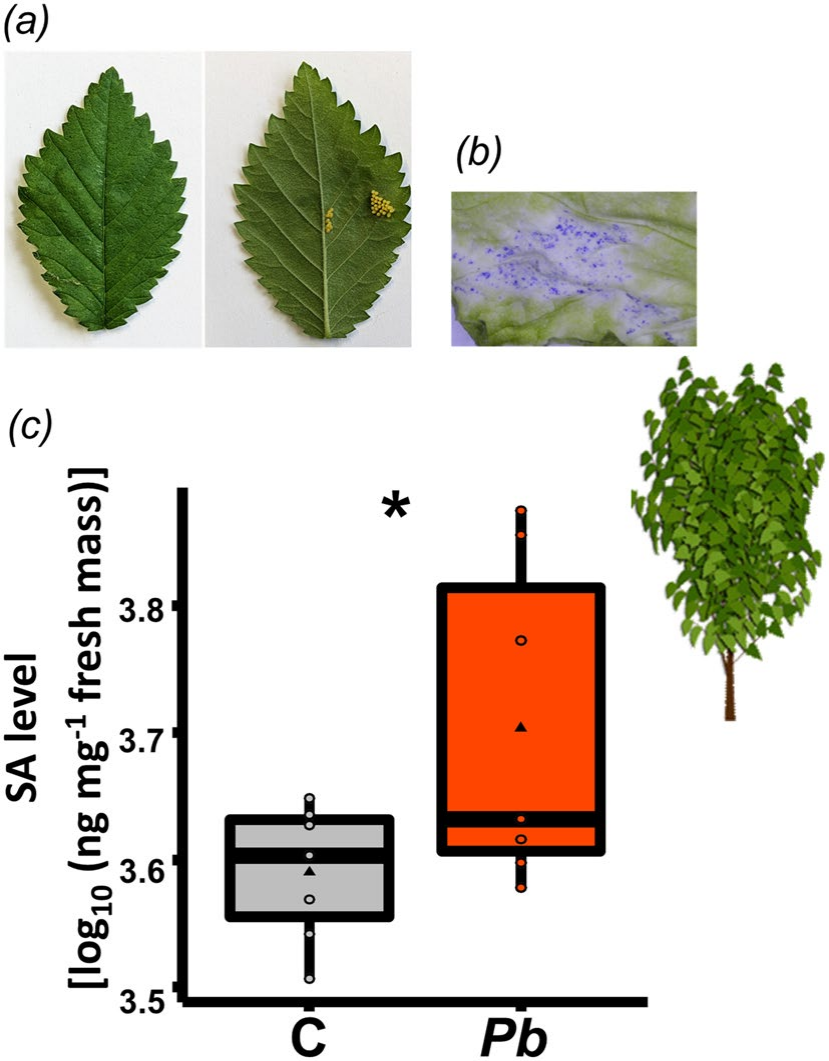
Response of *Ulmus minor* to *Pieris brassicae* egg deposition. (**a**) Elm leaves with *P. brassicae* eggs. (**b**) Cell death (detection with trypan blue) in leaves underneath the eggs. (**c**) Levels of salicylic acid (SA) in leaves three days after *P. brassicae* egg deposition (*Pb*) compared to untreated control (C) leaves. Boxplots show interquartile ranges (25–75%), medians (horizontal line), whiskers (10th and 90th percentiles) and means (triangles) of log_10_-transformed SA (ng per mg leaf fresh mass). Dots represent all data points. An asterisk (*) indicates a significant difference between the treatments (*P* < 0.05, Student’s *t*-test). *N* = 7. Statistical details are provided in Supplementary Table S5.

Leaves of *U. minor* did not show any chlorotic or necrotic tissue underneath the eggs three days after egg deposition (Fig. 5a). At this time point, treatment of leaves with DAB for detection of H_2_O_2_ did not result in stained tissue underneath the eggs. However, three days after egg deposition, trypan blue staining of *U. minor* leaves showed that this non-host plant also responds to *Pb* eggs by cell death (Fig. 5b). Overall, egg-free elm leaves had a very high SA level (Fig. 5). Despite of the already high SA level in egg-free *U. minor* leaves, *Pb* eggs still significantly induced SA levels in this plant (Fig. 5c, Supplementary Table S5).

Overall, when comparing the responses of *S. dulcamara* and *U. minor* to *Pb* eggs with the known response of *A. thaliana* and several other Brassicaceae to these eggs, the responses are similar with respect to the formation of leaf cell death and increase in SA concentrations^26,29,32,40,42^. Interestingly, one of the two genotypes of *S. dulcamara* showed a plant species- and even genotype-specific response to *Pb* eggs by the formation of neoplasms; no formation of neoplasms has so far been observed in other plant species in response to *Pb* eggs.

## Discussion

Our study on the insect species specificity of plant responses to insect eggs revealed that treatment of *A. thaliana* with egg extracts or egg secretion of a lepidopteran, a coleopteran and a hymenopteran species results in similar plant responses, but not always the same. Egg extracts of the three insect species induced SA concentrations in *A. thaliana.* Furthermore, the egg-associated secretions of the three insect species studied all induced *PR1, PR5* and *CAX3* in *A. thaliana.* Interestingly, all egg secretions contained PC derivatives, which have been shown before to induce *PR1* and SA levels in *A. thaliana*^45^. These findings suggest that egg secretions of different insect species share common plant response-eliciting compounds, among which PC derivatives seem to play a role; they might serve as general insect egg-associated molecular patterns, here referred to as EAMPs. PC derivatives are well known to affect membrane fluidity and elasticity, thus taking over numerous functional roles in most eukaryotes^51,52^. As components of insect egg-associated, exocrine secretions, they might serve as lubricants^53^ that facilitate the release of the eggs from the female's body.

Plant perception of insect-released PCs is known to elicit accumulation of ROS and SA and to induce defence gene expression and cell death in *A. thaliana*^45^*.* A lectin receptor kinase, LecRK-I.8, is suggested to be involved in perception of PCs. An *A. thaliana* mutant deficient in this kinase (*lecrk-I.8)* showed a much weaker response to *P. brassicae* eggs and to PCs than wild type plants^32,44,45,54^. Interestingly, *B. nigra* plants did not respond by cell death when treated with PCs^29^, and it is unclear whether this finding is due to a lack of a sufficiently sensitive receptor or other traits like e.g. the egg responsiveness of *B. nigra* sphingolipid biosynthesis genes^50^.

Many studies addressed the question how plants perceive danger-associated molecular patterns, such as microbe/pathogen-associated molecular patterns (MAMPs), herbivore-associated molecular patterns (HAMPS), or damage-induced molecular patterns (DAMPs)^27,55–59^. In addition to protein receptor-mediated recognition^59,60^, also the disturbance of the plant membrane architecture has been suggested to elicit defensive responses^61,62^. Several HAMPs, such as the fatty acid-amino acid conjugates released from oral secretions of different insect species, have an amphiphilic character that might elicit re-organization of the plasma membrane of a plant cell, resulting in depolarization of the membrane potential, eliciting a downstream defence signaling cascade^63^. Similarly, EAMPs like PCs or the bruchins released by bruchid beetles (C22–C24 α,ω-diols mono- or diesterified with 3-hydroxypropanoic acid^20^) have an amphiphilic character as well. Thus, it cannot be excluded so far that PCs present in insect egg secretions also act like a surfactant, thereby disturbing a plant's plasma membrane architecture and contributing to eliciting plant defence responses.

The finding that all the PC containing egg secretions of *Dp, Xl* and *Pb* induced expression of three well-known *Pb-*egg responsive marker genes in *A. thaliana* and other Brassicaceae^42,43,47–49^ suggests that these compounds are insect species-unspecific EAMPs, but the quantitative composition of the blend of PC isomers released by the insect during oviposition may vary between species. These results do not exclude that—in addition to the PCs—insect species-specific compounds in these secretions and/or the quantities of the released secretions elicit plant responses tailored to the respective species. In *Dp* egg secretion, an annexin-like protein named diprionin has been identified; this protein was shown to elicit an indirect defence response in the host plant of *Dp* by increasing the emission of a terpene that attracts egg parasitoids^64^ (Table 1). In *Pb* egg secretion, benzyl cyanide has been detected^65^; this compound elicits an indirect defence response in *A. thaliana* by changing the leaf surface chemistry as such that it becomes attractive to an egg parasitoid^18^ (Table 1). No defence-eliciting compounds have been identified thus far from *Xl* egg secretion. It remains to be studied how insect species-specific compounds in the egg secretions act in concert with the PCs.

The tested egg extracts showed both insect-species-specific and unspecific responses. Egg extracts of the three species induced SA concentrations, but camalexin levels were only induced by *Pb* and *Xl* egg extracts, but not by *Dp* egg extracts. Hence, the detection of insect species-specific and unspecific responses of *A. thaliana* to insect eggs depends on the type of egg material tested and on the response parameter considered.

When considering the plant species specificity of responses to insect eggs, our study showed that different plant species show similarities in their responses to *Pb* eggs. In addition to *A. thaliana,* also leaves of bittersweet nightshade (*S. dulcamara*) and elm (*U. minor*) responded to *Pb* eggs by increasing their SA levels, accumulating H_2_O_2_ and showing cell death at the site of egg deposition. These findings support the suggestion that plants show a phylogenetically conserved core response to insect eggs^28^. However, like for the insect species specificity, also plant species-specific responses to eggs were observed. For *A. thaliana,* even accession-specific responses to *Pb* eggs are known^44^. In our study, only *S. dulcamara* responded to *Pb* eggs by formation of neoplasms (Fig. 4a), while *A. thaliana* and *U. minor* showed no such response. *Solanum dulcamara* forms neoplasms also in response to eggs of the moth *Spodoptera exigua*^34^; hence, the formation of neoplasms in response to insect eggs is not highly insect species-specific in this plant species.

In summary, our study provides evidence that eggs and egg secretions from different insect species can elicit similar responses in *A. thaliana.* These findings suggest that insect eggs are associated with phylogenetically conserved, common plant response-eliciting EAMPs. PCs might represent such general EAMPs because (a) they were detected in egg secretions of the three species studied, (b) they are known to induce the SA level and *PR* gene expression^45^, (c) egg secretions of the three species studied also induced *PR* genes**,** and (d) egg extracts—known to contain PCs in *Pb*^45^—induced the SA level in *A. thaliana*. Insect species-specific compounds are suggested to further tune the plant response to the particular insect eggs laid on the leaves. Furthermore, we have shown here that very different plant species respond to *Pb* eggs with a similar core response. This core response is similar to that induced by (hemi)biotroph phytopathogens and includes an increase of the SA level and leaf cell death at the site where eggs are laid. This repertoire of plant species-independent responses may be complemented by plant species-specific responses, such as egg-induced formation of neoplasms in the case of *S. dulcamara.* Future studies need to clarify how plant and insect specific and unspecific traits interact in orchestrating plant responses to insect eggs.

## Methods

### Plants

For the experiments with *A. thaliana,* we used the ecotype Columbia-0 (Col-0). Seeds of *A. thaliana* Col-0 were obtained from the NASC-The European Arabidopsis Stock Centre. The plants germinated and grew on a 3:1 mixture of soil:vermiculite in climate chambers under short day conditions (8 h/16 h light dark cycle, 20 °C, 50% relative humidity and 100–120 µmol m^−2^ s^−1^ light intensity). For all experiments, six-week-old *A. thaliana* plants were used.

For the experiments with *S. dulcamara,* we used two different genotypes from Berlin Grunewald populations; genotype 1 was located at 52° 27′ 37.7″ N 13° 11′ 24.5″ E, genotype 2 was located at 52° 27′ 37.8″ N 13° 15′ 09.3″ E. Vouchers of this species are available at the locations mentioned here and at University Hohenheim, Germany, Molecular Botany Department. For our experiments we used three- to four-week-old plants grown from stem cuttings of five- to seven-week-old plants as described by Geuss et al*.*^34^. The plants grew under long day conditions (16 h/8 h light dark cycle, 23 °C, 70% relative humidity and 100–120 µmol m^−2^ s^−1^ light intensity).

We used ten-week-old *U. minor* trees, which were propagated in an in vitro tissue culture as described by Altmann et al*.*^66^. Tissue for growth of these trees was obtained from *U. minor* trees obtained from a tree nursery close to Berlin, Germany (www.Appel.Wald.de, Waldsieversdorf, Germany). When shoots had developed roots, the plants were transferred to soil. The plants grew under the abiotic conditions as described above for *S. dulcamara.*

### Insects

The butterfly *P. brassicae (Pb)* (Lepidoptera) was reared on Brussels sprouts as described by Valsamakis et al*.*^67^. The butterfly lays its eggs in a cluster on the lower side of host plant leaves (especially Brassicales); an egg cluster consists of 30–40 eggs and is attached to the leaf by secretion from the accessory reproductive gland of a female.

Larvae of the hymenopteran sawfly *D. pini* (*Dp)* were reared on the host plant Scots pine (*Pinus sylvestris* L.) as described by Bittner et al*.*^68^. The larvae are feeding gregariously on pine needles, while adults do not feed anymore. The females lay eggs in a row into a pine needle (here ~ 10–20 eggs per row), which is slit prior to oviposition by the female's sclerotized ovipositor for insertion of the eggs into the slit needle pouch. Each egg inside the needle is covered by a secretion from the female's *oviductus communis* (here referred to as "oviduct").

Adults of the elm leaf beetle *X. luteola* (*Xl)* were collected in the surroundings of Montpellier, France, and were kept on *U. minor* leaves for further rearing in the laboratory as described by Schott et al*.*^69^. Females lay eggs in a cluster (here ~ 10–20 eggs); they attach this cluster to the lower surface of elm leaves by a secretion from their oviduct. Prior to oviposition, they remove the leaf epidermis with their mouthparts at the site where they will deposit the eggs.

### Preparation of egg extracts and egg secretion samples

We used egg extracts and secretions for the treatment of *A. thaliana* because neither the sawfly *D. pini* nor the elm leaf beetle *X. luteola* could be motivated to naturally lay their eggs on this plant species. Furthermore, treatment of plants with egg extracts and secretions, respectively, allowed us (a) to check whether similar responses are inducible by these egg "materials" and (b) to treat the plant with comparable quantities of egg "materials".

For preparation of egg extracts, we removed *Dp* egg rows from pine needles, *Pb* egg clusters from *A. thaliana* leaves, and *Xl* egg clusters from elm leaves. For egg removal from the plants, we used a pair of tweezers and a fine brush. The eggs were transferred into 1.5 ml tubes. Eggs were crushed with a pestle and centrifuged at 13,000 rpm for 10 min at 4 °C. The supernatants were stored at − 80 °C similar as described by Bruessow et al*.*^42^. Crushing and centrifugation of *Xl* eggs did not yield a liquid supernatant; therefore, we omitted the centrifugation step and treated the plants with crushed eggs.

Egg secretion samples were obtained by dissecting the oviduct of *Dp* and *Xl* and the accessory reproductive gland (ARG) of *Pb* females. Secretions released from these organs are known to elicit defensive responses to the eggs laid onto the respective host plants^46,64,65^. For analysis of PCs, secretions from three oviducts and ARGs, respectively, were pooled to provide a sample. Samples used for plant treatments always contained secretion obtained from one female individual. Since males and females were kept together prior to collection of the secretion, females were most probably mated when choosing gravid females for the collection of their secretions. All samples were shock-frozen in liquid nitrogen and stored at -80 °C until used for plant treatments or analyses of PCs.

### Plant treatments and experiments

First, we addressed the question how insect species-specific the egg-induced response of *A. thaliana* is with respect to changes in concentrations of SA and camalexin and in expression of defence-related genes. We used egg extracts and egg-associated secretions to study this question. We measured SA and camalexin concentrations as well as expression of *A. thaliana* genes known to be responsive to *P. brassicae* eggs, i.e. we determined transcript levels of *PR1, PR5* and the cation exchanger gene *CAX3* ^47,48^.

Egg extract (2 µl) from *Dp* and *Pb*, respectively, was applied on the lower surface of one *A. thaliana* leaf in position 13–16 within the rosette. When testing *A. thaliana*'s response to *Xl* eggs, we applied 2 µg of crushed eggs to an *A. thaliana* leaf. After treatment, the plants were kept for 3 days under short day conditions (8 h/16 h light dark cycle, 20 °C, 65% relative humidity and 100–120 µmol m^−2^ s^−1^ light intensity) in a Percival. Prior to leaf sampling, the egg extracts were removed from the leaf using a fine brush. Thereafter, the entire previously extract-treated leaf was cut and transferred to a 2 ml screw cap tube. SA and camalexin were extracted and quantified as described below.

Egg-associated secretions were applied to the lower leaf surface of one *A. thaliana* leaf (a leaf in position 13–16 within the rosette). To test "female individual equivalents", we applied the entire volume of secretion from one *Dp* or *Xl* oviduct or from one *Pb* ARG to the leaf. Thereafter, plants were kept for three days in a Percival under the same short day conditions as mentioned above. Thereafter, the secretions were removed from the leaf with a smooth brush, and the entire leaf was harvested in order to quantify the expression of egg-responsive genes as described below.

Second, we investigated whether phosphatidylcholine derivatives (PCs) are present in the secretions associated with the eggs of the insect species studied, i.e., in oviduct secretion from *Dp* and *Xl* and ARG secretion from *Pb* (compare Table 1). PCs were extracted and quantified as described below.

Third, we analyzed the response of non-host plants to natural *Pb* egg deposition, thereby addressing the question how plant species-specific the responses are. We used elm (*U. minor*) and two genotypes of *S. dulcamara.* We succeeded in motivating *Pb* butterflies to lay eggs on the underside of leaves of these non-host plants by placing a host plant leaf (Brussels sprouts leaf) on top of a fully developed *S. dulcamara* leaf or an *U. minor* leaf, thus pretending a host plant substrate for oviposition. One female laid one egg cluster with 30–40 eggs per plant. The plants continued growing under long day conditions (16 h/8 h light dark cycle, 23 °C, 70% relative humidity and 100–120 µmol m^−2^ s^−1^ light intensity). We used different sets of plants treated this way for (1) observation of macroscopically visible, egg-induced leaf changes, (2) visualization of H_2_O_2_ accumulation and cell death, and (3) SA analysis. We used FlyInspecctor 30 (TechnoLab) to document macroscopically visible changes of the leaves in response to the eggs; photos were taken three and five days after egg deposition. To visualize H_2_O_2_ accumulation at the site of egg deposition by leaf staining, we gently removed the eggs from the leaves three days after egg deposition by using a fine brush and a pair of tweezers (for the staining method: see below). Stained leaves were photographed as well. For visualization of leaf cell death at the site of egg deposition, eggs were removed three days after egg deposition, and the oviposition site was stained (for the staining method: see below). For the analysis of SA in *S. dulcamara* and *U. minor*, we removed the *Pb* eggs three days after egg deposition and cut out leaf discs (Ø 15 cm) at the sites of the previous egg deposition; the discs were transferred in 2 ml screw cap tubes for analysis of locally egg-induced SA concentration.

### Leaf staining

Egg-treated *S. dulcamara* and *U. minor* leaves were stained with 3,3-diaminobenzidine (DAB) to visualize H_2_O_2_ accumulation and with lactophenol trypan blue solution to detect leaf cell death by staining^32^ (for further details, see Supplementary material: Methods).

### RNA extraction, cDNA synthesis and quantitative real-time PCR

Total RNA was extracted from untreated *A. thaliana* leaves and from leaves three days after application of egg secretion. The RNA extraction method followed the one described by Oñate-Sánchez and Vicente-Carbajosa^70^. Method details on cDNA synthesis and quantitative real-time PCR are provided in the Supplementary material: Methods. We calculated the relative expression of *PR1, PR5* and *CAX3* with the ∆∆CT method according to Livak and Schmittgen^71^. Two reference genes were used: *ACTIN 2, ACT2* (AT3G18780) and *POLYUBIQUITIN 10*, *UBQ10* (AT4G05320). The primers used are listed in Supplementary Table S6.

### Extraction and analysis of salicylic acid and camalexin from leaves

Leaf material from *A. thaliana*, *S. dulcamara* and *U. minor* was analyzed for SA concentrations. Additionally, camalexin concentrations were determined in leaf material from *A. thaliana.* SA and camalexin were extracted similar as described by Wang et al*.*^72^. We used untreated leaves, leaves locally treated for three days with egg extracts or leaves with natural egg deposition (for details on the extraction procedure, compare Supplementary material: Methods).

We used UPLC-MS/MS (Q-ToF-ESI; Synapt G2-S HDMS; Waters®, Milford, Massachusetts) for separation, detection and quantification of SA and camalexin. We applied the methods as described by Valsamakis et al*.*^40^ (further details in Supplementary material: Methods).

### Extraction and quantification of phosphatidylcholines in egg-associated secretions

Egg-associated secretion samples were subjected to lipid extraction using methanol/chloroform (2:1, v:v) as previously described for the extraction of diacylglycerols^73^. For absolute quantification of PC derivatives, the extraction solvent contained d_31_-16:0/18:1 PC as internal standard (Avanti Polar Lipids, Alabaster, USA).

Initially, lipid extracts were subjected to an untargeted HPLC–MS profiling of PC subspecies. To this end, a 1290 Infinity II HPLC coupled to a 6550 quadrupole time-of-flight (QTOF) mass spectrometer (both Agilent Technologies, Waldbronn, Germany) was used. Later, targeted quantifications of previously identified PC derivatives were performed using a set-up consisting of a 1290 Infinity II HPLC and a 6495 triple-quadrupole (QQQ) mass spectrometer (both Agilent Technologies). Method details on the chromatographic lipid separation is provided in Supplementary material: Methods. Ionization occurred in an electrospray interface operated in positive ion mode (ESI+). Ion source settings were adopted from our established method for quantification of sphingolipids^74^.

PC derivatives were identified using MassHunter Qualitative Analysis software (version 10.0, Agilent Technologies) with a mass inaccuracy of less than 5 ppm. Quantification of selected PC species, namely 16:1/16:1 PC, 18:1/18:1 PC and 18:3/18:3 PC, was performed on QQQ MS in multiple reaction monitoring (MRM) mode. Information on the recorded mass transitions is provided in Supplementary material: Methods; Fig. S1, Fig. S2.

Peak areas of PC derivatives were normalized to those of the internal standard (d_31_-16:0/18:1 PC) followed by external calibration in the range of 1 fmol to 50 pmol on column, using 16:1 (Δ9-*cis*) PC, 18:1 (Δ9-*cis*) PC and 18:3 (Δ9,12,15-*cis*) PC (all from Avanti Polar Lipids) as reference standards. Each sample contained secretions from three individuals. PC quantities were finally calculated per secretion extracted from one individual female.

### Data analysis and visualization

For statistical evaluation and visualization of data we used R (version 4.2.1)^75^ and R Studio (version 2022.07.1)^76^ and the following packages: car^77^, ggplot2^78^, psych^79^, Rmisc ^80^.

Distribution of the data was analyzed with Q-Q-plots and the Shapiro–Wilk test, the variance homogeneity was analyzed with the Levene’s test. For normally distributed data with homogenous variances, we applied Student’s *t*-test in case of dual comparison and ANOVA with Tukey test post hoc in case of multiple comparisons. The phytohormone and camalexin quantification data were log_10_ transformed to meet the criteria for a parametric test procedure. PC quantification data of the 18:1/18:1 and PC isomer 18:3/18:3 were left untransformed, because they met the criteria for a parametric test procedure (ANOVA with Tukey test post hoc); in case of the quantification data of the PC 16:1/16:1 isomer, data transformation did not result in normal distribution; therefore, these data were evaluated by a Kruskal–Wallis test. For the PC isomer 36:6 data, we did not apply any statistical test, because we did not detect this isomer in *Pb* and *Xl* secretions.

### Ethical standards

All our research complies with the relevant institutional, national, and international guidelines and legislation. No specific permissions or licenses are necessary for our research with the organisms studied here. Information on the identity of the plants is included in the “Methods” section, subsection ”Plants”; the mentioned companies guarantee for the correct identification of *A. thaliana* and *U. minor* as does the Molecular Botany Department at University Hohenheim, Germany, for the correct identification of *S. dulcamara.*

### Supplementary Information

Supplementary Information.

## Data Availability

In addition to data available in the manuscript and the "Supplementary Information" file (pdf), the gene expression datasets generated and analyzed during the current study are available online here: https://www.ebi.ac.uk/biostudies/studies/S-BSST1214?key=2cb63b90-53e4-442e-8803-13f744abe940.

## References

[1] Heil M, Bostock RM (2002). Induced systemic resistance (ISR) against pathogens in the context of induced plant defences. Ann. Bot..

[2] Chen M (2008). Inducible direct plant defense against insect herbivores: A review. Insect Sci..

[3] Dodds PN, Rathjen JP (2010). Plant immunity: Towards an integrated view of plant–pathogen interactions. Nat. Rev. Genet..

[4] Mithöfer A, Boland W (2012). Plant defense against herbivores: Chemical aspects. Annu. Rev. Plant Biol..

[5] War AR (2012). Mechanisms of plant defense against insect herbivores. Plant Signal. Behav..

[6] Huang X, Renwick JAA (1994). Cardenolides as oviposition deterrents to two *Pieris* species: Structure-activity relationships. J. Chem. Ecol..

[7] Katte T (2022). Oviposition stimulants underlying different preferences between host races in the leaf-mining moth *Acrocercops transecta* (Lepidoptera: Gracillariidae). Sci. Rep..

[8] Xing Z (2017). Efficiency of trichome-based plant defense in *Phaseolus vulgaris* depends on insect behavior, plant ontogeny, and structure. Front. Plant Sci..

[9] Hilker M, Fatouros NE (2015). Plant responses to insect egg deposition. Annu. Rev. Entomol..

[10] Hilker M, Fatouros NE (2016). Resisting the onset of herbivore attack: Plants perceive and respond to insect eggs. Curr. Opin. Plant Biol..

[11] Fatouros NE, Cusumano A, Danchin EGJ, Colazza S (2016). Prospects of herbivore egg-killing plant defenses for sustainable crop protection. Ecol. Evol..

[12] Reymond P (2013). Perception, signaling and molecular basis of oviposition-mediated plant responses. Planta..

[13] Reymond P (2022). The chemistry of plant–insect egg interactions. Chimia..

[14] Hilker M, Salem H, Fatouros NE (2023). Adaptive plasticity of insect eggs in response to environmental ehallenges. Annu. Rev. Entomol..

[15] Hilker M, Meiners T (2006). Early herbivore alert: Insect eggs induce plant defense. J. Chem. Ecol..

[16] Hilker M, Meiners T (2011). Plants and insect eggs: How do they affect each other?. Phytochemistry..

[17] Hilker M, Meiners T (2002). Induction of plant responses to oviposition and feeding by herbivorous arthropods: A comparison. In Entomol. Exp. Appl..

[18] Blenn B (2012). Insect egg deposition induces indirect defense and epicuticular wax changes in *Arabidopsis thaliana*. J. Chem. Ecol..

[19] Fatouros NE (2005). Oviposition-induced plant cues: Do they arrest *Trichogramma* wasps during host location?. Entomol. Exp. Appl..

[20] Doss RP (2000). Bruchins: Insect-derived plant regulators that stimulate neoplasm formation. Proc. Natl. Acad. Sci. U.S.A..

[21] Petzold-Maxwell J, Wong S, Arellano C, Gould F (2011). Host plant direct defence against eggs of its specialist herbivore, *Heliothis subflexa*. Ecol. Entomol..

[22] Shapiro AM, DeVay JE (1987). Hypersensitivity reaction of *Brassica nigra* L. (Cruciferae) kills eggs of *Pieris butterflies* (Lepidoptera: Pieridae). Oecologia..

[23] Balbyshev NF, Lorenzen JH (1997). Hypersensitivity and egg drop: A novel mechanism of host plant resistance to Colorado potato beetle (Coleoptera: Chrysomelidae). J. Econ. Entomol..

[24] Fatouros NE (2014). Synergistic effects of direct and indirect defences on herbivore egg survival in a wild crucifer. Proc. R. Soc. B..

[25] Griese E, Dicke M, Hilker M, Fatouros NE (2017). Plant response to butterfly eggs: Inducibility, severity and success of egg-killing leaf necrosis depends on plant genotype and egg clustering. Sci. Rep..

[26] Griese E (2021). Insect egg-killing: A new front on the evolutionary arms-race between brassicaceous plants and pierid butterflies. New Phytol..

[27] Jones AC, Felton GW, Tumlinson JH (2022). The dual function of elicitors and effectors from insects: Reviewing the ‘arms race’ against plant defenses. Plant Mol. Biol..

[28] Lortzing T, Kunze R, Steppuhn A, Hilker M, Lortzing V (2020). Arabidopsis, tobacco, nightshade and elm take insect eggs as herbivore alarm and show similar transcriptomic alarm responses. Sci. Rep..

[29] Caarls L (2023). Hypersensitive-like response in *Brassica* plants is specifically induced by molecules from egg-associated secretions of cabbage white butterflies. Front. Ecol. Evol..

[30] Schott J, Fuchs B, Böttcher C, Hilker M (2022). Responses to larval herbivory in the phenylpropanoid pathway of *Ulmus minor* are boosted by prior insect egg deposition. Planta..

[31] Kim J, Tooker JF, Luthe DS, Moraes D, Felton CM (2012). Insect eggs can enhance wound response in plants: A study system of tomato *Solanum lycopersicum* L. and *Helicoverpa zea* Boddie. PLoS One..

[32] Gouhier-Darimont C, Schmiesing A, Bonnet C, Lassueur S, Reymond P (2013). Signalling of *Arabidopsis thaliana* response to *Pieris brassicae* eggs shares similarities with PAMP-triggered immunity. J. Exp. Bot..

[33] Bittner N, Trauer-Kizilelma U, Hilker M (2017). Early plant defence against insect attack: Involvement of reactive oxygen species in plant responses to insect egg deposition. Planta..

[34] Geuss D, Stelzer S, Lortzing T, Steppuhn A (2017). *Solanum dulcamara*’s response to eggs of an insect herbivore comprises ovicidal hydrogen peroxide production. Plant Cell Environ..

[35] Balint-Kurti P (2019). The plant hypersensitive response: Concepts, control and consequences. Mol. Plant Pathol..

[36] Torres MA, Dangl JL, Jones JDG (2002). Arabidopsis gp91phox homologues AtrbohD and AtrbohF are required for accumulation of reactive oxygen intermediates in the plant defense response. Proc. Natl. Acad. Sci. U.S.A..

[37] Vlot AC, Dempsey DA, Klessig DF (2009). Salicylic acid, a multifaceted hormone to combat disease. Annu. Rev. Phytopathol..

[38] Hilfiker O (2014). Insect eggs induce a systemic acquired resistance in Arabidopsis. Plant J..

[39] Bednarek P (2012). Chemical warfare or modulators of defence responses—The function of secondary metabolites in plant immunity. Curr. Opin. Plant Biol..

[40] Valsamakis G (2020). Priming by timing: *Arabidopsis thaliana* adjusts its priming response to Lepidoptera eggs to the time of larval hatching. Front. Plant Sci..

[41] Alfonso E (2021). Insect eggs trigger systemic acquired resistance against a fungal and an oomycete pathogen. New Phytol..

[42] Bruessow F, Gouhier-Darimont C, Buchala A, Métraux J-P, Reymond P (2010). Insect eggs suppress plant defence against chewing herbivores. Plant J..

[43] Paniagua Voirol LR (2020). Plant responses to insect eggs are not induced by egg-associated microbes, but by a secretion attached to the eggs. Plant Cell Environ..

[44] Groux R (2021). Arabidopsis natural variation in insect egg-induced cell death reveals a role for LECTIN RECEPTOR KINASE-I.1. Plant Physiol..

[45] Stahl E (2020). Phosphatidylcholines from *Pieris brassicae* eggs activate an immune response in arabidopsis. Elife..

[46] Meiners T, Hilker M (2000). Induction of plant synomones by oviposition of a phytophagous insect. J. Chem. Ecol..

[47] Lortzing V (2019). Insect egg deposition renders plant defense against hatching larvae more effective in a salicylic acid-dependent manner. Plant Cell Environ..

[48] Little D, Gouhier-Darimont C, Bruessow F, Reymond P (2007). Oviposition by pierid butterflies triggers defense responses in Arabidopsis. Plant Physiol..

[49] Bonnet C (2017). Combined biotic stresses trigger similar transcriptomic responses but contrasting resistance against a chewing herbivore in *Brassica nigra*. BMC Plant Biol..

[50] Groux R, Fouillen L, Mongrand S, Reymond P (2022). Sphingolipids are involved in insect egg-induced cell death in Arabidopsis. Plant Physiol..

[51] Smaby JM, Momsen MM, Brockman HL, Brown RE (1997). Phosphatidylcholine acyl unsaturation modulates the decrease in interfacial elasticity induced by cholesterol. Biophys. J..

[52] Bao X (2021). Shortening of membrane lipid acyl chains compensates for phosphatidylcholine deficiency in choline-auxotroph yeast. Embo J..

[53] Hill K (2000). Fats and oils as oleochemical raw materials. Pure Appl. Chem..

[54] Gouhier-Darimont C, Stahl E, Glauser G, Reymond P (2019). The Arabidopsis lectin receptor kinase LecRK-I.8 is involved in insect egg perception. Front. Plant Sci..

[55] Mithöfer A, Boland W (2008). Recognition of herbivory-associated molecular patterns. Plant Physiol..

[56] Bonaventure G (2012). Perception of insect feeding by plants. Plant Biol..

[57] Heil M (2012). How plants sense wounds: Damaged-self recognition is based on plant-derived elicitors and induces octadecanoid signaling. PLoS One..

[58] Boutrot F, Zipfel C (2017). Function, discovery, and exploitation of plant pattern recognition receptors for broad-spectrum disease resistance. Annu. Rev. Phytopathol..

[59] Snoeck S, Guayazán-Palacios N, Steinbrenner AD (2022). Molecular tug-of-war: Plant immune recognition of herbivory. Plant Cell..

[60] Wang J, Chai J (2020). Structural insights into the plant immune receptors PRRs and NLRs. Plant Physiol..

[61] Sandor R (2016). Plasma membrane order and fluidity are diversely triggered by elicitors of plant defence. J. Exp. Bot..

[62] Schellenberger S (2019). Highly fluorinated chemicals in functional textiles can be replaced by re-evaluating liquid repellency and end-user requirements. J. Clean. Prod..

[63] Spiteller D, Dettner K, Boland W (2000). Gut bacteria may be involved in interactions between plants, herbivores and their predators: Microbial biosynthesis of *N*-acylglutamine surfactants as elicitors of plant volatiles. Biol. Chem..

[64] Hundacker J (2022). Pine defense against eggs of an herbivorous sawfly is elicited by an annexin-like protein present in egg-associated secretion. Plant Cell Environ..

[65] Fatouros NE (2008). Male-derived butterfly anti-aphrodisiac mediates induced indirect plant defense. Proc. Natl. Acad. Sci. USA..

[66] Altmann S (2018). Transcriptomic basis for reinforcement of elm antiherbivore defence mediated by insect egg deposition. Mol. Ecol..

[67] Valsamakis G, Bittner N, Kunze R, Hilker M, Lortzing V (2022). Priming of Arabidopsis resistance to herbivory by insect egg deposition depends on the plant’s developmental stage. J. Exp. Bot..

[68] Bittner N, Hundacker J, Achotegui-Castells A, Anderbrant O, Hilker M (2019). Defense of Scots pine against sawfly eggs (*Diprion pini*) is primed by exposure to sawfly sex pheromones. Proc. Natl. Acad. Sci. U.S.A..

[69] Schott J, Jantzen F, Hilker M (2023). Elm tree defences against a specialist herbivore are moderately primed by an infestation in the previous season. Tree Physiol..

[70] Oñate-Sánchez L, Vicente-Carbajosa J (2008). DNA-free RNA isolation protocols for *Arabidopsis thaliana*, including seeds and siliques. BMC Res. Notes..

[71] Livak KJ, Schmittgen TD (2001). Analysis of relative gene expression data using real-time quantitative PCR and the 2(− delta delta C(T)) method. Methods..

[72] Wang L (2007). Independently silencing two JAR family members impairs levels of trypsin proteinase inhibitors but not nicotine. Planta..

[73] Zeitler S (2020). Acid sphingomyelinase impacts canonical transient receptor potential channels 6 (TRPC6) activity in primary neuronal systems. Cells..

[74] Naser E (2020). Characterization of the small molecule ARC39, a direct and specific inhibitor of acid sphingomyelinase in vitro. J. Lipid Res..

[75] R Core Team. R Development Core Team. *R A Lang. Environ. Stat. Comput.* https://www.r-project.org/ (2016).

[76] R Studio Team. *RStudio: Integrated Development for R.* RStudio, Inc., Boston, MA (Computer Software v0.98.1074). http://www.rstudio.com/ (2015).

[77] Fox J, Weisberg S (2019). R Companion to Applied Regression.

[78] Wickham, H. *ggplot2—Positioning Elegant Graphics for Data Analysis*. (2016).

[79] Revelle, W. *psych: Procedures for Personality and Psychological Research*. https://CRAN.R-project.org/package=psych (2020).

[80] Hope, R. *CRAN—Package Rmisc.* (accessed 2023). https://cran.r-project.org/web/packages/Rmisc/index.html (2022).

[81] Fatouros NE (2009). Anti-aphrodisiac compounds of male butterflies increase the risk of egg parasitoid attack by inducing plant synomone production. J. Chem. Ecol..

[82] Yang JO, Nakayama N, Toda K, Tebayashi S, Kim CS (2013). Elicitor(s) in *Sogatella furcifera* (Horvath) causing the Japanese rice plant (*Oryza sativa* L.) to induce the ovicidal substance, benzyl benzoate. Biosci. Biotechnol. Biochem..

[83] Yang JO, Nakayama N, Toda K, Tebayashi S, Kim CS (2014). Structural determination of elicitors in *Sogatella furcifera* (Horváth) that induce Japonica rice plant varieties (*Oryza sativa* L.) to produce an ovicidal substance against *S. furcifera* eggs. Biosci. Biotechnol. Biochem..

