# Supplementary material for: Plant defensive responses to insect eggs are inducible by general egg-associated elicitors
Source: Sci Rep. 2024 Jan 11;14:1076. doi: 10.1038/s41598-024-51565-y (PMC10784483; doi:10.1038/s41598-024-51565-y)
Supplement: Supplementary file 1 — Supplementary Information. [file 41598_2024_51565_MOESM1_ESM.pdf]

Plant defensive responses to insect eggs are inducible by general egg-associated elicitors

Vivien Lortzing[1], Georgios Valsamakis[1], Friederike Jantzen[1], Janik Hundacker[1], Luis R. Paniagua Voirol[1,2], Fabian Schumacher[3, 4], Burkhard Kleuser[3] and Monika Hilker[1]

[1] Applied Zoology / Animal Ecology, Institute of Biology, Dahlem Centre of Plant Sciences, Freie Universität Berlin, Haderslebener Str. 9, 12163 Berlin, Germany

[2] Microbiology, Institute of Biology, Dahlem Centre of Plant Sciences , Freie Universität Berlin, Königin-Luise-Str. 12-16, 14195 Berlin, Germany

[3] Pharmacology and Toxicology, Institute of Pharmacy, Freie Universität Berlin, Königin-Luise-Str. 2-4, 14195 Berlin, Germany

[4] Core-Facility BioSupraMol, PharmaMS subunit, Institute of Pharmacy, Freie Universität Berlin, Königin-Luise-Str. 2-4, 14195 Berlin, Germany

Supplementary Material: Overview

Supplementary Tables

Table S1: Statistical details on phytohormone and camalexin data presented in Fig. 1.

Table S2. Statistical details on gene expression data presented in Fig. 2.

Table S3. Statistical details on phosphatidylcholine data in Fig. 3.

Table S4. Statistical details on salicylic acid data presented in Fig. 4.

Table S5. Statistical details on salicylic acid data presented in Fig. 5.

Table S6. Analyzed genes and qRT-primers.

Supplementary Figures

Figure S1. Multiple reaction monitoring (MRM) chromatograms of selected phosphatidylcholines (PCs) in egg-associated secretions of different insect species.

Figure S2. Identification of the 36:6 PC isomer 2 found in oviduct secretion of *Diprion pini* (*Dp*).

Figure S3. Response of *Solanum dulcamara* genotype 2 in response to 2 µl of *Pieris brassicae* egg extract.

Supplementary Material: Methods

- Leaf staining
- cDNA synthesis and quantitative real-time PCR
- Extraction and analysis of salicylic acid and camalexin from leaves
- Analysis of phosphatidylcholines in egg-associated secretions

Supplementary Tables

Table S1. Statistical details on phytohormone and camalexin data presented in Fig. 1.

Salicylic acid: ANOVA with Tukey *post hoc* test

	Df	Sum Sq	Mean Sq	F value	Pr(>F)	
dataframe$treatment	3	8.033	2.6777	32.08	3.36E-09	***
Residuals	28	2.337	0.0835			

	diff	Lwr	upr	p adj
Dp-C	1.18674173	0.79235243	1.581131	0
Pb-C	0.49079192	0.09640262	0.8851812	0.0104386
Xl-C	1.18414788	0.78975858	1.5785372	0
Pb-Dp	-0.6959498	-1.0903391	-0.3015605	0.0002538
Xl-Dp	-0.0025939	-0.3969832	0.3917954	0.9999979
Xl-Pb	0.69335596	0.29896666	1.0877453	0.0002664

Level (mean ± SE) in ng*mg^{-1} fresh weight:

	mean		SE
C	9.65	±	0.93
Dp	157.39	±	27.23
Pb	54.45	±	24.67
Xl	163.24	±	39.05

Camalexin: ANOVA with Tukey *post hoc* test

	Df	Sum Sq	Mean Sq	F value	Pr(>F)
dataframe$treatment	3	11.3	3.768	132	<2e-16
Residuals	28	0.8	0.029		

	diff	lwr	upr	p adj
Dp-C	-0.1258064	-0.3564961	0.1048834	0.4572934
Pb-C	0.2518945	0.02120476	0.4825842	0.0283516
Xl-C	1.3782895	1.14759975	1.6089792	0
Pb-Dp	0.3777009	0.14701113	0.6083906	0.0006463
Xl-Dp	1.5040959	1.27340612	1.7347856	0
Xl-Pb	1.126395	0.89570525	1.3570847	0

Level (mean ± SE) in ng*mg^{-1} fresh weight:

	mean		SE
C	181.45	±	47.7
Dp	117.41	±	8.05
Pb	291.65	±	38.75
Xl	3920.22	±	495.67

Abbreviations: C: untreated control; *Dp: Dprion pini* egg extract, *Pb: Pieris brassicae* egg extract; *Xl: Xanthogaleruca luteola* egg extract

Table S2. Statistical details on gene expression data presented in Fig. 2. The data were statistically evaluated with ANOVA and Tukey *post hoc* test.

CAX3					
	Df	Sum Sq	Mean Sq	F value	Pr(>F)
dataframe$treatment	3	76.8	25.6	11.84	0.000674
Residuals	12	25.96	2.163		
	diff	lwr	upr	p adj	
Dp-C	4.5898236	1.502257	7.67739	0.0040552	
Pb-C	5.7936154	2.706049	8.881182	0.0006077	
Xl-C	4.2824299	1.194863	7.369997	0.0067357	
Pb-Dp	1.2037918	-1.883775	4.291359	0.6630587	
Xl-Dp	-0.3073937	-3.39496	2.780173	0.9905506	
Xl-Pb	-1.5111855	-4.598752	1.576381	0.4928744	
PR1					
	Df	Sum Sq	Mean Sq	F value	Pr(>F)
dataframe$treatment	3	221.1	373.7	11.59	0.000738
Residuals	12	076.3	6.36		
	diff	lwr	upr	p adj	
Dp-C	8.144048	2.850554	13.437542	0.0031217	
Pb-C	9.4771853	4.183691	14.77068	0.0009114	
Xl-C	7.7487034	2.455209	13.042198	0.0045503	
Pb-Dp	1.3331373	-3.960357	6.626632	0.8759082	
Xl-Dp	-0.3953446	-5.688839	4.89815	0.9959398	
Xl-Pb	-1.7284819	-7.021976	3.565012	0.7688676	
PR5					
	Df	Sum Sq	Mean Sq	F value	Pr(>F)
dataframe$treatment	3	84.67	28.224	11.34	0.000813
Residuals	12	29.87	2.489		
	diff	lwr	upr	p adj	
Dp-C	4.1236427	0.8114054	7.43588	0.0140355	
Pb-C	6.4036906	3.0914533	9.715928	0.000467	
Xl-C	3.8698594	0.5576221	7.182097	0.0209108	
Pb-Dp	2.2800479	-1.0321894	5.592285	0.226115	
Xl-Dp	-0.2537833	-3.5660207	3.058454	0.9956212	
Xl-Pb	-2.5338313	-5.8460686	0.778406	0.1596409	

Abbreviations: C: untreated control; *Dp: Dprion pini* egg secretion, *Pb: Pieris brassicae* egg secretion; *Xl: Xanthogaleruca luteola* egg secretion

Table S3. Statistical details on phosphatidylcholine data in Fig. 3.

**PC 16:1/16:1: Kruskal-Wallis rank sum test**					
Kruskal-Wallis chi-squared = 5.0667, df = 2, p-value = 0.07939					
**PC 18:1/18:1: ANOVA and Tukey *post hoc* test**					
	Df	Sum Sq	Mean Sq	F value	Pr(>F)
dataframe$treatment	2	21041	10521	20.36	0.00212
Residuals	6	3100	517		
	diff	lwr	upr	p adj	
Pp-Dp	-103.859347	-160.80368	-46.91501	0.0033450	
Xl-Dp	-2.630141	-59.57448	54.31420	0.9890091	
Xl-Pb	101.229207	44.28487	158.17354	0.0038099	
**PC 18:3/18:3: ANOVA and Tukey *post hoc* test**					
	Df	Sum Sq	Mean Sq	F value	Pr(>F)
dataframe$treatment	2	45672	22836	18.99	0.00254
Residuals	6	7214	1202		
	diff	lwr	upr	p adj	
Pp-Dp	158.73053	71.86290	245.59816	0.0033134	
Xl-Dp	142.13194	55.26431	228.99957	0.0057613	
Xl-Pb	-16.59859	-103.46623	70.26904	0.8322677	

Table S4. Statistical details on salicylic acid data presented in Fig. 4.

**Genotype 1:** Student's *t*-tests			
t = -9.8515	df = 18	p-value = 1.124e-08	
Level (mean ± SE) in ng*mg^{-1} fresh weight:			
	mean		SE
C	16.35	±	1.03
Pb	120.73	±	23.12
**Genotype 2:** Student's *t*-tests			
t = -2.4195	df = 14	p-value = 0.02974	
Level (mean ± SE) in ng*mg^{-1} fresh weight:			
	mean		SE
C	41.68	±	10.53
Pb	75.52	±	12.77

Abbreviations: C: untreated control; *Pb: Pieris brassicae* eggs

Table S5. Statistical details on salicylic acid data presented in Fig. 5.

**Salicylic acid:** Student's *t*-tests			
t = -2.1836	df = 12	p-value = 0.04957	
Level (mean ± SE) in ng*mg^{-1} fresh weight:			
	mean		SE
C	3915.34	±	175.55
Pb	5245.13	±	599.18

Abbreviations: C: untreated control; *Pb: Pieris brassicae* eggs

Table S6. Analyzed genes and qRT-primers designed by PerlPrimer (Marshall 2004) and obtained from Eurofins Genomics.

		**Sequence 5´--> 3´**	
**Target**	**AGI**	**Forward**	**Reverse**
ACT2	AT3G18780	CTTCCCTCAGCACATTCCAG	GACCTGCCTCATCATACTCG
UBQ10	AT4G05320	GGCCTTGTATAATCCCTGATGAATAAG	AAAGAGATAACAGGAACGGAAACATAGT
CAX3	AT3G51860	CTTTCTACACTGGTCCAACAGTG	TATTTCACCACTGCCACTTTGTTA
PR1	AT2G14610	ACACGTGCAATGGAGTTTGTGG	TTGGCACATCCGAGTCTCACTG
PR5	AT1G75040	GATGTGAGCCTCGTAGATGGT	ACATTGTTCTGATCCATGACCT

Marshall, OJ. PerlPrimer: cross-platform, graphical primer design for standard, bisulphite and real-time PCR. *Bioinformatics* **20**, 2471-2472. https://doi.org/10.1093/bioinformatics/bth254 (2004).

Supplementary Figure S1

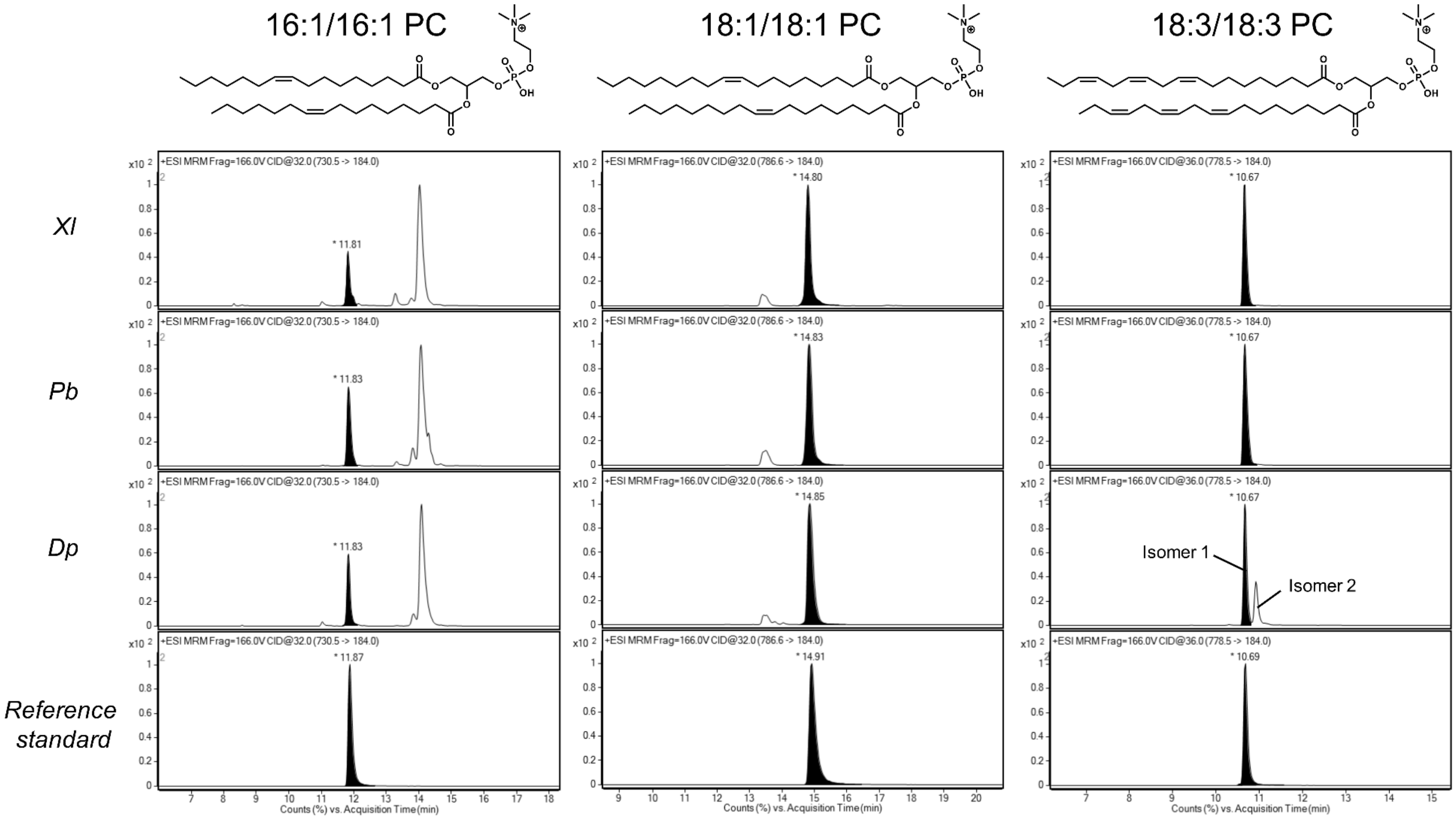

Figure S1. **Multiple reaction monitoring (MRM) chromatograms of selected phosphatidylcholines (PCs) in egg-associated secretions of different insect species.** In *Xanthogaleruca luteola* (*Xl*) and *Diprion pini* (*Dp*), secretions were taken from oviducts. In *Pieris brassicae* (*Pb*), secretions were taken from female accessory reproductive glands. For the identification of PC subspecies, reference substances were analyzed separately, the chemical structure of which is given at the very top. Shown are only the quantifier mass transitions $[M+H]^+ \rightarrow m/z$ 184. All black coloured signals showed two further qualifier mass transitions in the previously optimized intensity ratios. Peaks are labeled with retention time. +ESI, positive mode electrospray ionization; Frag, fragmentor voltage, CID, collision-induced dissociation.

Supplementary Figure S2

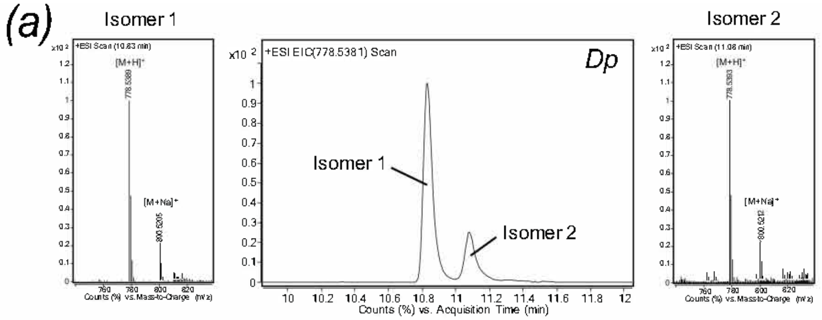

Formula PC 36:6 $[M+H]^+$: $C_{44}H_{77}NO_8P$

Exact mass $[M+H]^+$ (*m/z*): 778.5381

Mass error $[M+H]^+$ (Isomer 1): 1.03 ppm

Mass error $[M+H]^+$ (Isomer 2): 1.54 ppm

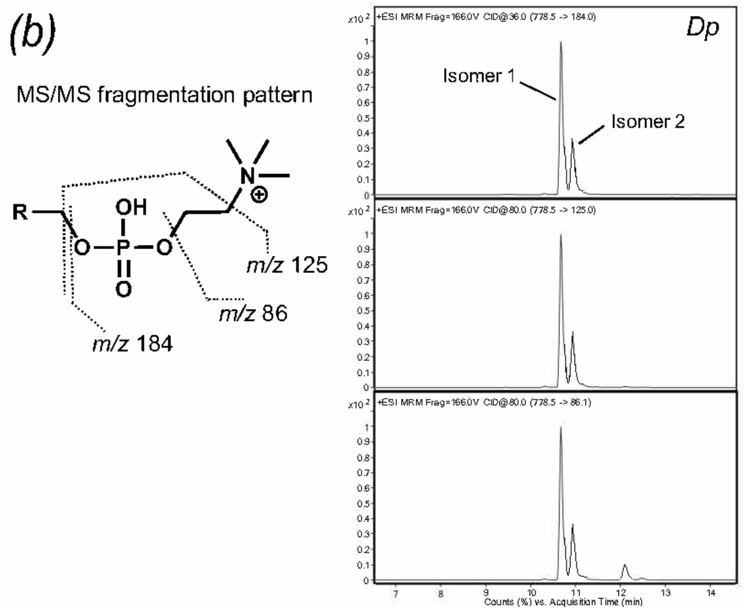

(c) Lipid Maps ® search

Filter: precursor ion: *m/z* 778.5393
type of ion adduct: $[M+H]^+$
mass tolerance: +/- 0.01 *m/z*
lipid classes: phosphatidylcholines and sphingomyelins

Lipid Maps ® ID	Common Name	
LMGP01010512	PC(14:0/22:6(4Z,7Z,10Z,13Z,16Z,19Z))	
LMGP01012134	PC(14:1(9Z)/22:5(4Z,7Z,10Z,13Z,16Z))	
LMGP01012135	PC(14:1(9Z)/22:5(7Z,10Z,13Z,16Z,19Z))	
LMGP01011492	PC(16:1(9Z)/20:5(5Z,8Z,11Z,14Z,17Z))	
LMGP01011627	PC(18:2(9Z,12Z)/18:4(6Z,9Z,12Z,15Z))	
LMGP01011654	PC(18:3(6Z,9Z,12Z)/18:3(6Z,9Z,12Z))	
LMGP01011655	PC(18:3(6Z,9Z,12Z)/18:3(9Z,12Z,15Z))	
LMGP01010952	PC(18:3(9E,11E,13E)/18:3(9E,11E,13E))	
LMGP01010954	PC(18:3(9Z,11E,13E)/18:3(9Z,11E,13E))	
LMGP01011684	PC(18:3(9Z,12Z,15Z)/18:3(6Z,9Z,12Z))	
LMGP01010956	PC(18:3(9Z,12Z,15Z)/18:3(9Z,12Z,15Z))	→ Isomer 1
LMGP01011713	PC(18:4(6Z,9Z,12Z,15Z)/18:2(9Z,12Z))	
LMGP01010960	PC(18:4(9E,11E,13E,15E)/18:2(9Z,12Z))	
LMGP01011933	PC(20:5(5Z,8Z,11Z,14Z,17Z)/16:1(9Z))	
LMGP01012233	PC(22:5(4Z,7Z,10Z,13Z,16Z)/14:1(9Z))	
LMGP01012235	PC(22:5(7Z,10Z,13Z,16Z,19Z)/14:1(9Z))	
LMGP01012099	PC(22:6(4Z,7Z,10Z,13Z,16Z,19Z)/14:0)	

Figure S2. Identification of the 36:6 PC isomer 2 found in oviduct secretion of *Diprion pini* (*Dp*). *(a)* HPLC coupled to high-resolution mass spectrometry showed two signals eluting close to each other, which may be assigned to the molecular formula $C_{44}H_{77}NO_8P$ (e.g., $[M+H]^+$ of PC 36:6) due to high mass accuracy ($\Delta m/z$ less than 1.6 ppm). This is supported by the fact that isomer 1 could be identified as 18:3/18:3 PC by means of a reference standard. *(b)* Isomer 1 and 2 exhibit three MS/MS fragmentations characteristic for a phosphocholine group in the molecule. This observation further narrows the possible lipid class for isomer 2, basically to PCs and sphingomyelins. *(c)* A Lipid Maps® search (https://www.lipidmaps.org/) yielded a total of 17 possible structures with the listed search criteria for the specific exact mass-to-charge ratio of isomer 2 (*m/z* 778.5393). One of these is PC 18:3(9Z,12Z,15Z)/18:3(9Z,12Z,15Z), which could already be assigned to isomer 1 using a reference standard.

Supplementary Figure S3

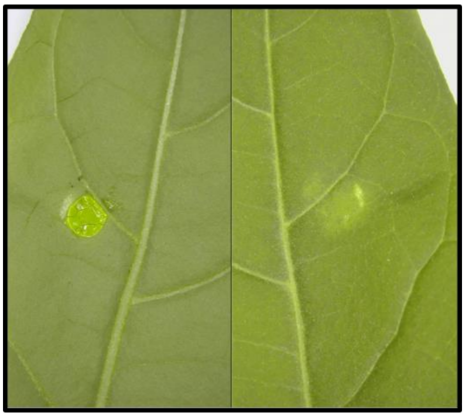

Figure S3. **Response of *Solanum dulcamara* genotype 2 in response to 2 µl of *Pieris brassicae* egg extract.** Left: lower leaf surface with egg extract; right: View on upper leaf surface with chlorotic spot formed at the site treated with egg extract.

Supplementary Material: Methods

Method: Leaf staining

We stained leaves three days after removal of the eggs with a 1.0 mg ml^{-1} 3,3-diaminobenzidine (DAB) solution to visualize H_2O_2 accumulation at the site of previous egg deposition. The leaves were submerged in the DAB solution and incubated in the dark at room temperature for 7 h.

Leaf cell death underneath the eggs was visualized by staining the leaves also three days after egg deposition with lactophenol trypan blue solution [5 ml of lactic acid, 10 ml of 50% glycerol, 2 ml of 0.4% trypan blue and 5 ml of phenol]. The leaves were submerged at 30°C for 3 h. Each leaf was distained in boiling 95% ethanol for 10 min.

Method: cDNA synthesis and quantitative real-time PCR

After RNA extraction, residue genomic DNA was digested with the DNA-free™ DNA Removal Kit (Thermo Fisher Scientific, Life Technologies). The first strand cDNA was synthesized from 2 µg RNA with the RevertAid First Strand cDNA synthesis kit (Thermo Fisher Scientific) and oligo-dT18 following the manufacturer´s protocol. We analyzed the expression of *PR1, PR5* and *CAX3.* The quantitative real-time PCRs were conducted in 10 µl reactions using Power SYBR® Green PCR master mix (Applied Biosystems, Life technologies) as described by Valsamakis *et al.* (2022).

Valsamakis,G., Bittner, N., Kunze, R., Hilker, M. & Lortzing, V. Priming of Arabidopsis resistance to herbivory by insect egg deposition depends on the plant's developmental stage. *J. Exp. Bot.* **73**, 4996–5015. (https://doi.org/10.1093/JXB/ERAC199 (2022).

Method: Extraction and analysis of salicylic acid and camalexin from leaves

For quantification of salicylic acid and camalexin concentrations in *A. thaliana* leaves, the leaf material was homogenized in 2 ml tubes with beats (Zirconox, 2.8-3.3 mm, Mühlmeier Mahltechnik, Bärnau, Germany) in the FastPrep®-24 instrument (MP Biomedicals, Solon, USA) at 6 m s^{-1} for 20 s. We added 500 to 1000 µl ethyl acetate and 2 µl d_4-SA internal standard (10 ng μl^{-1}) (OlChemIm Ltd., Olomouc, Czech Republic). Samples were again homogenized in ethyl acetate 2 x 20 s at 6 m s^{-1}. Samples were centrifuged for 10 min at 4°C and 13.000 rpm using an Eppendorf® centrifuge 5427R with rotor FA-45-48-11 (Eppendorf AG, Hamburg, Germany). The extraction was repeated with pure ethyl acetate. The supernatants of the two extraction steps were combined. After vaporization of the supernatant with the Eppendorf Concentrator 5301 vacuum concentrator, SA and camalexin were re-eluted in 300 µl 70% methanol with 0.1% formic acid (v/v) under 10 min vortexing at room temperature. The samples were centrifuged for 15 min at 13,000 rpm and room temperature. Finally, 200 µl of the supernatant were transferred to glass vials.

By applying UPLC-MS/MS analysis, salicylic acid was quantified according to peak areas of the fragment ions relative to the fragment ion of the d_4-SA internal standard. Camalexin was quantified with an external standard curve using following dilution series: 0 µM, 0.1 µM, 0.5 µM, 1, 5 µM, 7.5 µM, 10 µM, 50 µM [M (camalexin) = 200.26 g·mol^{-1}].

Method: Analysis of phosphatidylcholines in egg-associated secretions

Egg-associated secretions of the three insect species studied were subjected to chromatographic lipid separations for both untargeted QTOF and targeted QQQ analysis.

The separations were conducted on a Poroshell 120 EC-C8 column (3.0 x 150 mm, 2.7 µm; Agilent Technologies). A mobile phase system consisting of water (solvent A) and acetonitrile/methanol (1:1, v:v; solvent B), both acidified with 0.1% formic acid, was used for gradient elution at an initial composition of 40:60 (A:B, v:v) and a flow rate of 0.5 ml min^{-1}. The column was kept at 30°C, and the injection volume was 10 µl.

QTOF analysis was performed in scan mode in the range of *m/z* 50-1,500 with a scan rate of 2 spectra. The reference masses *m/z* 121.0509 and *m/z* 922.0098 were continuously supplied by an isocratic pump.

Quantification of selected PC species was performed on QQQ MS in multiple reaction monitoring (MRM) mode. The following mass transitions were recorded (collision energies in parentheses): *m/z* 730.5 → 184.0 (32 eV)/ 125.0 (76 eV)/ 86.1 (80 eV) for 16:1/16:1 PC, *m/z* 778.5 → 184.0 (36 eV)/ 125.0 (80 eV)/ 86.1 (80 eV) for 18:3/18:3 PC, *m/z* 786.6 → 184.0 (32 eV)/ 125.0 (80 eV)/ 86.1 (80 eV) for 18:1/18:1 PC and *m/z* 791.8 → 184.0 (38 eV)/ 125.0 (80 eV)/ 86.1 (80 eV) for d_{31}-16:0/18:1 PC (compare figures S1, S2). The dwell time for all mass transitions recorded was 70 ms. Collision-induced dissociations yielding the phosphocholine group ($[M+H]^+$ → *m/z* 184.0) served as quantifiers.
